# Spark Plasma Sintering of Pristine and Transition Metal-Doped Ti_2_AlC MAX Phases

**DOI:** 10.3390/ma18091957

**Published:** 2025-04-25

**Authors:** Mikhail S. Gurin, Dmitry S. Shtarev, Ilya A. Zavidovskiy, Erkhan S. Kolodeznikov, Andrey A. Vyshnevyy, Aleksey V. Arsenin, Alexey D. Bolshakov, Alexander V. Syuy

**Affiliations:** 1 Department of Nuclear Technologies, Far Eastern Federal University, Vladivostok 690922, Russia; gurin.ms@dvfu.ru (M.S.G.); kolodeznikov.es@dvfu.ru (E.S.K.); 2 Department of Materials Science, Shenzhen MSU-BIT University, Shenzhen 518115, China; 3 Moscow Center for Advanced Studies, Kulakova str. 20, Moscow 123592, Russia; ia.zavidovskii@physics.msu.ru (I.A.Z.); vyshnevyy@xpanceo.com (A.A.V.); arsenin@xpanceo.com (A.V.A.); bolshakov@live.com (A.D.B.); 4 Emerging Technologies Research Center, XPANCEO, Dubai, United Arab Emirates; 5 Laboratory of Advanced Functional Materials, Yerevan State University, Yerevan 0025, Armenia; 6 Center for Nanotechnologies, Alferov University, Saint Petersburg 194021, Russia; 7 Faculty of Physics, St. Petersburg State University, Saint Petersburg 199034, Russia; 8 Depatrment of Physics, Perm National Research Polytechnic University, Perm 614990, Russia

**Keywords:** spark plasma synthesis, MAX phase, transition metal doping, Ti_2_AlC purity

## Abstract

We study the synthesis of Ti_2_AlC MAX-phase ceramics via spark plasma sintering (SPS), focusing on the effects of temperature, precursor composition, and transition metal doping (Mo, Ta, Hf, W, Y, and Mn). Optimized sintering parameters were established, defining the precursor ratios necessary for the formation of Ti_2_AlC with >90% yield. Structural and compositional analyses revealed that select transition metals—Ta, Hf, W, and Y—could be incorporated into the Ti_2_AlC lattice, which resulted in >90% yield for each transition metal-doped MAX phase. In contrast, Mo and Mn predominantly formed separate phases. These findings provide insights into the controlled synthesis of MAX-phase materials with tunable properties for high-performance applications.

## 1. Introduction

MAX phases are a unique group of materials with a distinctive structure and a blend of useful properties. These ternary carbides and nitrides have the general formula M_n+1_AX_n_, where M denotes an early transition metal, A is an element from groups 13–16, and X is carbon or nitrogen. Their dual metallic and ceramic-like behavior has attracted considerable research interest [1,2]. Thanks to their high corrosion resistance, thermal stability, flexural strength, and fracture toughness, they show great potential for various applications [3,4,5,6,7].

MAX phases derive their structural stability from a nanolayered crystal structure with a hexagonal unit cell (space group P6_3_/*mmc*). In this configuration, MX octahedra alternate with A-atom layers, enabling good electrical and thermal conductivity while maintaining mechanical strength. Growing interest in these materials has led to various synthesis methods, such as electrochemical and hydrothermal approaches, sol-gel techniques, and chemical vapor deposition (CVD). Currently, sintering methods are most widely used because they are simple to implement, based on established powder metallurgy practices, and work with readily available starting materials [8].

Different sintering methods each have their own strengths and weaknesses. Self-propagating high-temperature synthesis is simple to perform but produces granular materials because no pressure is applied during formation [9]. Cold sintering is relatively easy to implement but requires long processing times [10]. Microwave sintering encounters difficulties with uneven power distribution and requires specialized equipment [11]. In contrast, spark plasma sintering (SPS) offers several advantages that overcome these limitations. SPS allows for fast heating, reduced processing times, improved energy efficiency, and better control over microstructure with fine grain sizes [8]. Importantly, this technique can consolidate powders at temperatures below their melting points, significantly accelerating production [12].

Sintering methods also enable the synthesis of doped MAX phases by replacing precursor materials with selected dopants [13], which can significantly alter material properties. Transition metal (TM) doping shows particular promise, as it may improve mechanical strength, corrosion resistance, catalytic activity, and even introduce magnetic properties at appropriate doping concentrations [14,15,16]. These enhancements make TM-doped MAX phases suitable for a wider range of applications.

Among these materials, TM-doped Ti_2_AlC has attracted special attention due to its tunable mechanical [17] and corrosion properties [15] along with adjustable thermal stability [18]. However, synthesizing TM-doped MAX phases remains challenging because high electron counts typical for transition metals often lead to structural instability and the formation of competing thermodynamic phases [19]. Understanding the feasibility and limitations of TM doping is, therefore, essential for expanding MAX-phase applications into new fields.

This study systematically investigates three critical parameters in Ti_2_AlC MAX-phase synthesis via spark plasma sintering: (1) sintering temperature, (2) precursor stoichiometry, and (3) transition metal doping (Mo, Ta, Hf, W, Y, and Mn). Additionally, we investigate the purity of the resultant materials and quantify the maximum incorporation thresholds for each dopant within the Ti_2_AlC lattice. The resulting framework enables precise engineering of pristine and TM-doped MAX-phase materials.

## 2. Materials and Methods

### 2.1. Sintering Temperature Optimisation

Undoped Ti_3_AlC_2_ was synthesized using titanium carbide (TiC), metallic titanium (Ti), and aluminum carbide (Al_4_C_3_) as precursor materials. The precursors were first dry-ground in stoichiometric ratios required for Ti_3_AlC_2_ formation to ensure homogeneous mixing. Mechanochemical processing was then performed using a ball mill operating at 300 rpm with a cyclic regime of 20 min grinding, followed by 10 min pauses. The milling media consisted of zirconia oxide (ZrO_2_) vials and balls, employing a bimodal distribution of ball sizes: larger balls (5–7 mm diameter) and smaller balls (1–1.2 mm diameter). This size distribution was selected to optimize grinding efficiency through enhanced kinetic energy transfer and sufficient mixing. The total active mixing time was 10 h (15 h including pauses), using a ball-to-powder mass ratio of 1:10 throughout the process. This two-stage preparation method yielded uniformly mixed precursor powders ready for subsequent sintering. Ball-milling was carried out in ambient conditions. The homogenized powders were consolidated using spark plasma sintering (SPS) in a Dr. Sinter·LAB™ SPS-515S system (Fuji Electronic Industrial Co., Tokyo, Japan). For each trial, 3 g of powder was loaded into a 15 mm graphite mold, pre-pressed at 20.7 MPa, and sintered under vacuum (10^−5^ atm). The SPS process employed a unipolar low-voltage pulsed current in On/Off mode (12 pulses/2 pauses, 39.6 ms pulse duration, 6.6 ms pause duration). Temperature was monitored via an optical pyrometer (detection threshold: 650 °C) focused on a 5.5 mm deep window in the mold wall. Graphite foil (200 μm thickness) prevented powder adhesion to mold components, while heat-insulating fabric minimized thermal losses. The resulting cylindrical samples measured 15 mm in diameter with heights varying between 4–10 mm depending on sintering conditions. To evaluate temperature effects, powders were consolidated at 1200 °C, 1300 °C, and 1400 °C using a staged heating protocol: 300 °C/min (0–650 °C) followed by 50 °C/min (>650 °C). The samples were held at target temperatures for 10 min before 30 min cooling to room temperature. The resulting specimens were designated as Ti(7):TiC(5):Al_4_C_3_(1)-1200, Ti(7):TiC(5):Al_4_C_3_(1)-1300, and Ti(7):TiC(5):Al_4_C_3_(1)-1400 (see sintering details in Table S1). Here and in subsequent notations, numbers in parentheses denote the molar ratios of the corresponding components.

### 2.2. Optimisation of Excess Aluminium Content for Ti_2_AlC Synthesis

To investigate the effect of aluminum excess on phase formation, Ti_2_AlC was synthesized with controlled additions of aluminum carbide (Al_4_C_3_). Three distinct compositions were prepared by systematically varying the Al_4_C_3_ content at 10 mol.%, 30 mol.%, and 50 mol.% above the stoichiometric requirement, enabling a direct comparison of how aluminum enrichment influences the yield of the target MAX phase. The powders were subsequently consolidated via spark plasma sintering (SPS) using experimental procedures similar to those described in Section 2.1, maintaining a constant sintering temperature of 1300 °C. The resulting samples with varying aluminum carbide excesses were designated as Ti(7):TiC(1):Al_4_C_3_(1.1), Ti(7):TiC(1):Al_4_C_3_(1.3), and Ti(7):TiC(1):Al_4_C_3_(1.5), corresponding to 10 mol%, 30 mol%, and 50 mol% Al_4_C_3_ excess, respectively (see synthesis parameters in Table S2).

### 2.3. Synthesis of Doped MAX Phases

To synthesize transition metal-doped MAX phases, we employed partial substitution of titanium in the Ti_2_AlC precursor mixture with selected dopants (Mo, Ta, HfC, W, Y, and Mn). The substitution levels were systematically varied up to the maximum incorporation limit of each transition metal in the MAX-phase structure. The doped samples were then synthesized following the same spark plasma sintering protocol described in Section 2.1, ensuring consistent processing conditions across all of the specimens. Samples obtained with 10 and 20 mol.% molybdenum doping are denoted as Ti(6.3):TiC(1):Al_4_C_3_(1.3):Mo(0.7) and Ti(5.6):TiC(1):Al_4_C_3_(1.3):Mo(1.4) (see synthesis conditions in Table S3), respectively. MAX phases doped with 10, 20, 30, and 40 mol.% tantalum are denoted as Ti(6.3):TiC(1):Al_4_C_3_(1.3):Ta(0.7), Ti(5.6):TiC(1):Al_4_C_3_(1.3):Ta(1.4), Ti(4.9):TiC(1):Al_4_C_3_(1.3):Ta(2.1), and Ti(3.5):TiC(1):Al_4_C_3_(1.3):Ta(2.5), respectively (see sintering protocols in Table S4). Materials doped with 10 and 20 mol.% Hf are named Ti(6.3):TiC(1):Al_4_C_3_(1.3):HfC(0.7) and Ti(5.6):TiC(1):Al_4_C_3_(1.3):HfC(1.4), respectively (see preparation details in Table S5). Samples doped with 10 and 20 mol.% Hf are denoted as Ti(6.3):TiC(1):Al_4_C_3_(1.3):W(0.7) and Ti(5.6):TiC(1):Al_4_C_3_(1.3):W(1.4), respectively (see fabrication parameters in Table S6). Materials doped with 10 and 20 mol.% yttrium are named Ti(6.3):TiC(1):Al_4_C_3_(1.3):Y(0.7) and Ti(5.6):TiC(1):Al_4_C_3_(1.3):Y(1.4), respectively (see sintering conditions in Table S7). Samples doped with 10 and 20 at.% manganese are denoted as Ti(6.3):TiC(1):Al_4_C_3_(1.3):Mn(0.7) and Ti(5.6):TiC(1):Al_4_C_3_(1.3):Mn(1.4) (see synthesis details in Table S8).

### 2.4. Sample Characterization

The phase composition of the synthesized materials was characterized using powder X-ray diffraction (PXRD) performed on a Bruker D8 Advance diffractometer (Bruker Optik GmbH, Ettlingen, Germany) equipped with a Vantec-1 linear detector. Measurements were conducted using Cu Kα₁ radiation (λ = 1.5406 Å) generated at 40 kV voltage and 15 mA current of cathod ray tube, with data collected over a 2θ range of 5–62° using a step size of 0.02° and an accumulation time of 35.4 s per point. The sample morphology and elemental composition were analyzed using a SCIOS 2 scanning electron microscope (Thermo Fisher Scientific, Waltham, MA, USA) equipped with an energy-dispersive X-ray spectroscopy (EDS) detector.

## 3. Results

### 3.1. The Effect of Temperature on the Yield of the Target Phase

**Figure 1:**
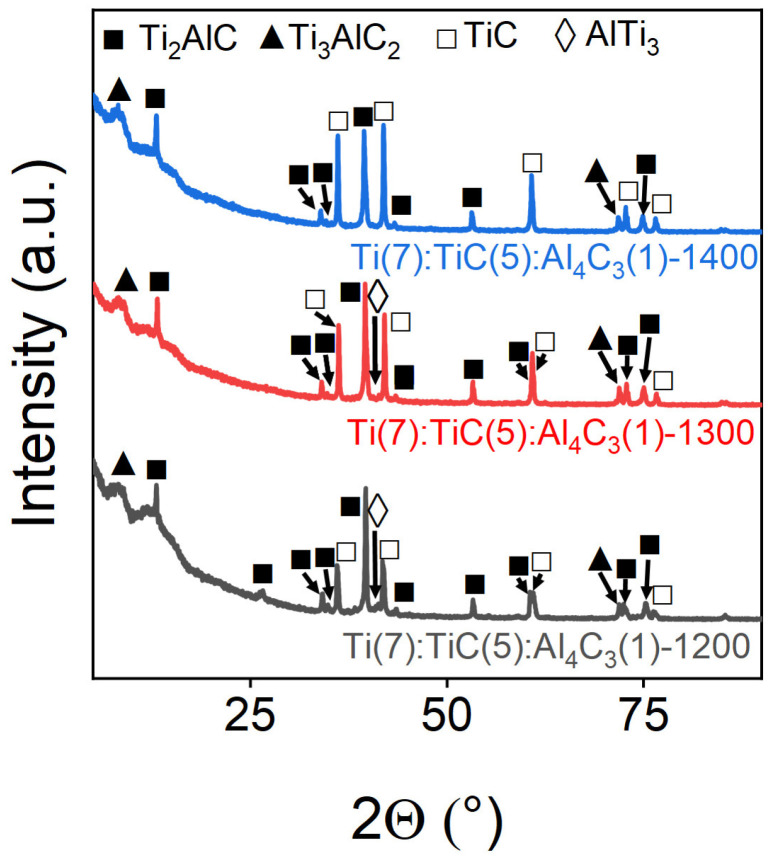
Powder X-ray diffraction patterns of Ti(7):TiC(5):Al_4_C_3_(1)-1200/1300/1400 samples of a stoichiometric Ti_3_AlC_2_ composition obtained at various sintering temperatures. The marks above the reflections indicate the corresponding target and impurity phases.

The influence of the sintering temperature on the Ti_3_AlC_2_ MAX-phase formation and impurity content was investigated across the 1200–1400 °C range. Figure 1 presents the PXRD patterns of the resulting samples. At 1200 °C, the Ti_3_AlC_2_ phase formation was incomplete, as evidenced by weak diffraction peaks relative to the coexisting Ti_2_AlC and TiC phases. Increasing the temperature to 1300 °C enhanced the 312-phase formation, with significantly intensified Ti_3_AlC_2_ diffraction peaks. However, at 1400 °C, sample outflow from graphite molds occurred during processing. Notably, all samples retained TiC and Ti_2_AlC phase reflections, suggesting insufficient energy for complete conversion to the Ti_3_AlC_2_ phase under these conditions.

**Figure 2:**
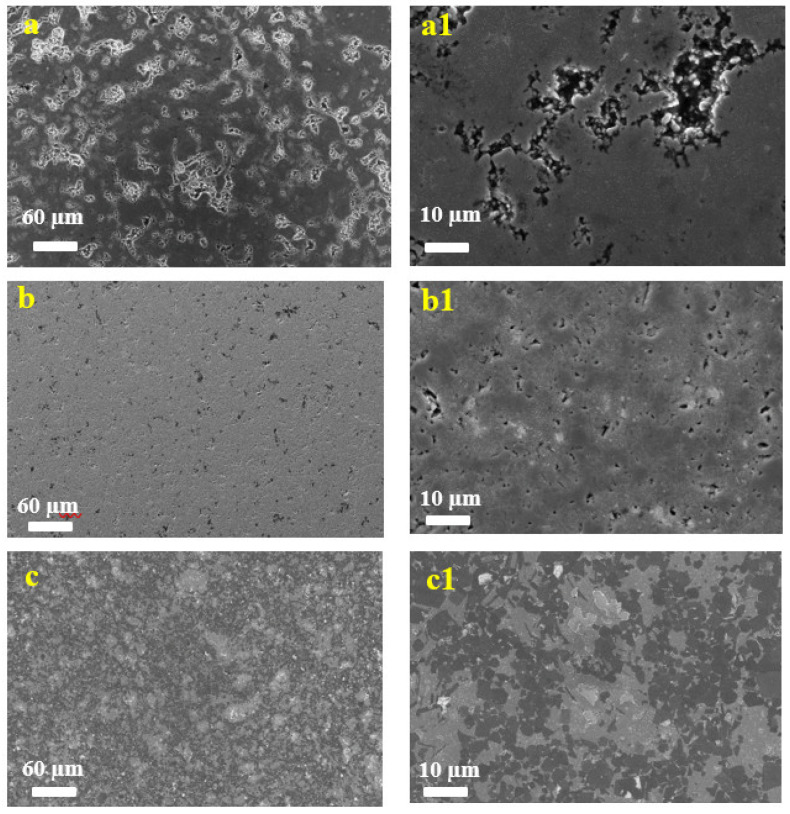
SEM images of Ti(7):TiC(5):Al_4_C_3_(1)-1200/1300/1400 samples obtained at various temperatures: 1200 °C (**a**,**a1**), 1300 °C (**b**,**b1**), and 1400 °C (**c**,**c1**).

Figure 2 presents the surface morphology of the samples synthesized at different temperatures. The micrographs reveal distinct structural evolution with increasing temperature: samples processed at 1300 °C and 1400 °C exhibit prominent surface scaling. The textured microstructure becomes more pronounced at higher sintering temperatures, suggesting temperature-dependent grain growth.

**Figure 3:**
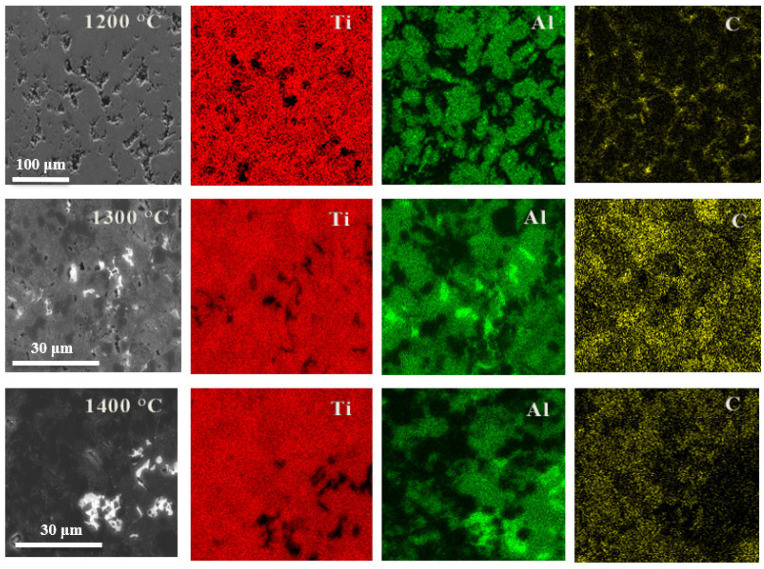
SEM images of Ti(7):TiC(5):Al_4_C_3_(1)-1200 (first row)/1300 (second row)/1400 (third row) samples obtained at various temperatures and mapping of the distribution of various elements obtained by X-ray energy dispersion spectroscopy mapping. SEM images (first column) and EDS mappings of Ti (second column), Al (third column), and C (fourth column) are presented.

While PXRD analysis confirmed the presence of both target MAX phases and secondary phases, their distinct elemental compositions enable spatial distribution analysis through SEM-EDS elemental mapping. Figure 3 demonstrates this phase segregation, revealing the heterogeneous distribution of constituent elements across the sample microstructure.

EDS mapping of the 1200 °C sintered sample revealed distinct elemental distribution patterns: titanium appeared uniformly dispersed while aluminum formed localized aggregates (Figure 3). This segregation indicates an incomplete reaction between the constituent elements, leaving significant amounts of unreacted titanium primarily in the form of TiC. These observations correlate well with the PXRD results in Figure 1, which show strong TiC reflections alongside weaker MAX-phase peaks. The 1300 °C sintered samples exhibited the most homogeneous distribution of Ti, Al, and C among all of the specimens, consistent with optimal MAX-phase formation, as confirmed by PXRD (Figure 1). Nevertheless, EDS mapping revealed residual Ti- and C-rich regions devoid of Al, corresponding to unreacted TiC precursor particles. These isolated domains, while less prevalent than in the 1200 °C samples, demonstrated that even at this sintering temperature, the reaction remained incomplete. An EDS analysis of the 1400 °C sintered samples revealed distinct Al-rich regions lacking both Ti and C, suggesting the formation of metallic aluminum phases. This phase separation, not observed at lower sintering temperatures, indicates partial decomposition of the MAX-phase structure under these high-temperature conditions.

Table 1 presents the measured atomic ratios of constituent elements. The elemental composition analysis revealed a significant sintering temperature dependence influence on the final elemental ratios. All of the synthesized samples exhibited non-stoichiometric compositions, particularly showing titanium and carbon excess alongside aluminum deficiency. The 1300 °C sample demonstrated the closest approximation to the 3:1:2 (Ti:Al:C) stoichiometry. We attribute the systematic aluminum deficit primarily to the volatilization of aluminum-containing species during spark plasma sintering, consistent with the lower thermal stability of aluminum carbide. Based on these observations, we investigated aluminum-enriched precursor compositions as a potential route to improve the MAX-phase yield.

### 3.2. The Effect of the Precursor Ratio on the Yield of Ti_2_AlC

The results in Section 3.1 demonstrate that sintering stoichiometric 312-phase precursor mixtures yielded substantial amounts of the 211-phase (Ti_2_AlC), with the 211-phase content increasing systematically with temperature, as evidenced by the growing intensity of the characteristic PXRD reflections. This temperature-dependent phase competition indicates that the available energy remained insufficient for the complete conversion of precursors to the Ti_3_AlC_2_ phase across the investigated temperature range. The change in aluminum-containing precursor ratio is a conventional way to modify the phase composition and stoichiometry of sintered MAX phases [20]. Thus, to optimize the Ti_2_AlC yield, we systematically investigated aluminum carbide excess in the precursor mixtures (10, 30, and 50 mol% beyond stoichiometry).

**Figure 4:**
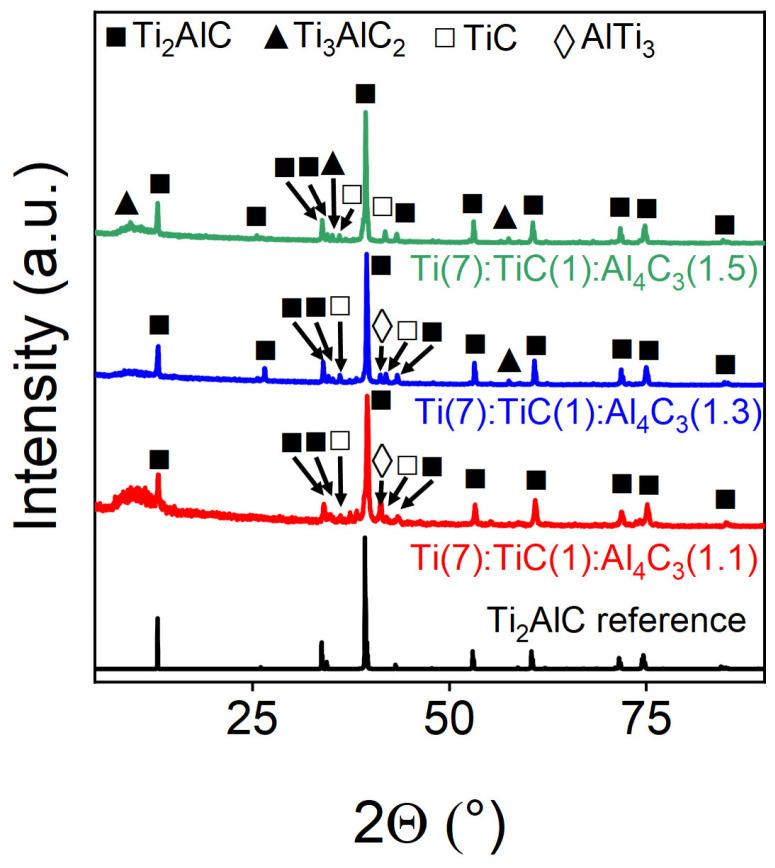
Experimental powder X-ray diffraction patterns of Ti(7):TiC(1):Al_4_C_3_(1.1/1.3/1.5) samples obtained at different concentrations of super stoichiometric aluminum. The marks above the reflections indicate the corresponding target and impurity phases.

XRD analysis (Figure 4) revealed that adding 30% of Al_4_C_3_ produced Ti_2_AlC phase of the highest purity. At a lower excess (10%), increased AlTi_3_ intermetallic phase formation was observed, while higher excess (50%) promoted partial conversion to the Ti_3_AlC_2_ phase instead.

Quantitative phase analysis via Rietveld refinement (Table 2) confirmed that introducing 30 mol% excess aluminum carbide significantly enhanced the target phase yield, achieving >90% Ti_2_AlC purity. This optimal aluminum excess effectively suppressed the secondary phase formation while maximizing the desired MAX-phase content.

### 3.3. The Effect of Doping Additives on MAX-Phases

To elaborate on the possibility of doped MAX-phase synthesis via SPS, optimal conditions allowing for 90.32% Ti_2_AlC synthesis were modified through the addition of transition metals.

**Figure 5:**
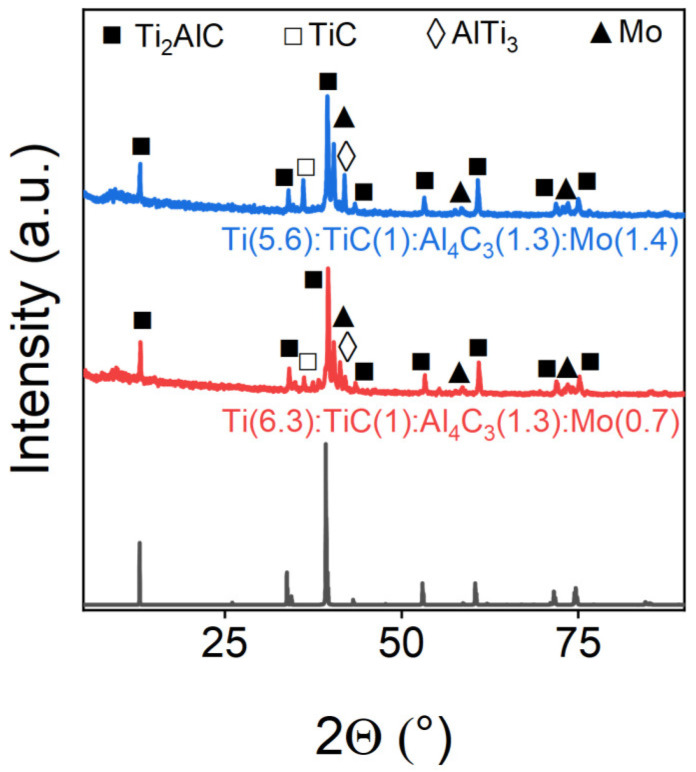
Powder X-ray diffraction patterns of Mo-doped samples. The marks above the reflections indicate the corresponding target and impurity phases.

Building on the optimized synthesis conditions that yielded 90.32% pure Ti_2_AlC, we investigated transition metal doping via SPS. Figure 5 presents the XRD patterns of Mo-doped samples, revealing minimal molybdenum incorporation into the MAX-phase structure. Distinct metallic Mo peaks appeared in the diffraction patterns, with Rietveld refinement (Table 3) quantitatively confirming the predominant presence of metallic molybdenum rather than the formation of the doped MAX phase.

**Figure 6:**
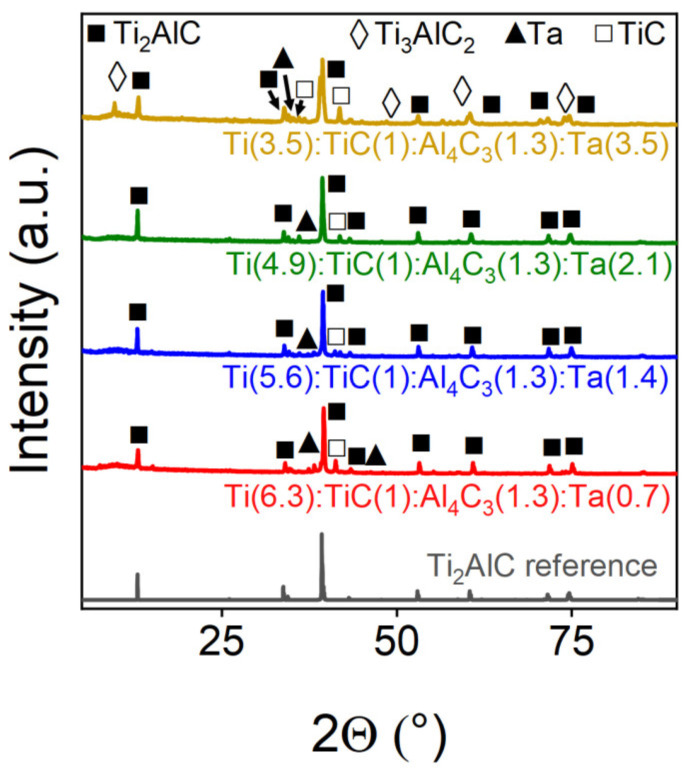
Powder X-ray diffraction patterns of Ta-doped samples. The marks above the reflections indicate the corresponding target and impurity phases.

In contrast with molybdenum, tantalum demonstrated effective incorporation into the Ti_2_AlC MAX-phase structure (Figure 6, Table 4). Samples containing up to 30% Ta achieved an approximately 90% target phase yield, with tthe increasing Ta content correlating with enhanced phase purity. Remarkably, replacing 50% of titanium with tantalum enabled synthesis of the Ti_3_AlC_2_ 312-phase (~50% yield). However, Rietveld analysis (Table 4) revealed that high Ta concentrations led to mixed-phase products containing both 211 and 312 MAX-phases along with metallic Ta and TiC, suggesting the need for further optimization of Ta-doped MAX-phase synthesis via SPS.

**Figure 7:**
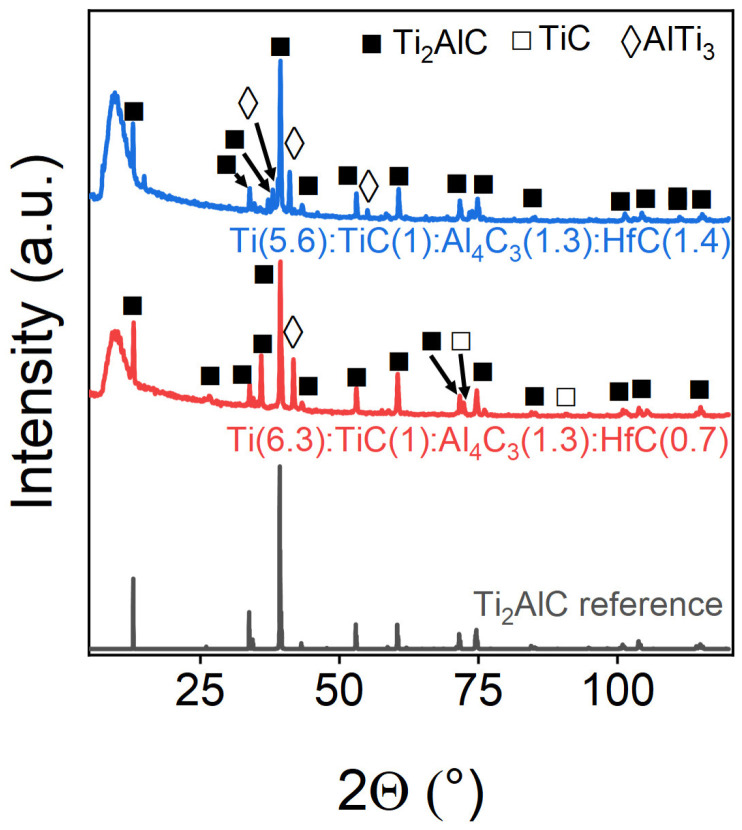
Powder X-ray diffraction patterns of Hf-doped samples. The marks above the reflections indicate the corresponding target and impurity phases.

Hafnium demonstrates effective but limited incorporation into the MAX-phase structure. XRD analysis (Figure 7) and corresponding Rietveld refinement data (Table 5) revealed complete hafnium integration at 10% doping levels. However, increasing the hafnium content to 20% generated additional diffraction peaks between 35–38° (2θ), suggesting the formation of secondary phases—likely metallic hafnium or hafnium carbide (HfC). This threshold behavior indicates a solubility limit for hafnium in the MAX phase under these synthesis conditions. While hafnium fully integrates into the crystal structure, we observed a concomitant 20% increase in AlTi_3_ intermetallic phase content compared with undoped Ti_2_AlC. This suggests that Hf doping may promote secondary phase formation even while maintaining the MAX-phase structure.

**Figure 8:**
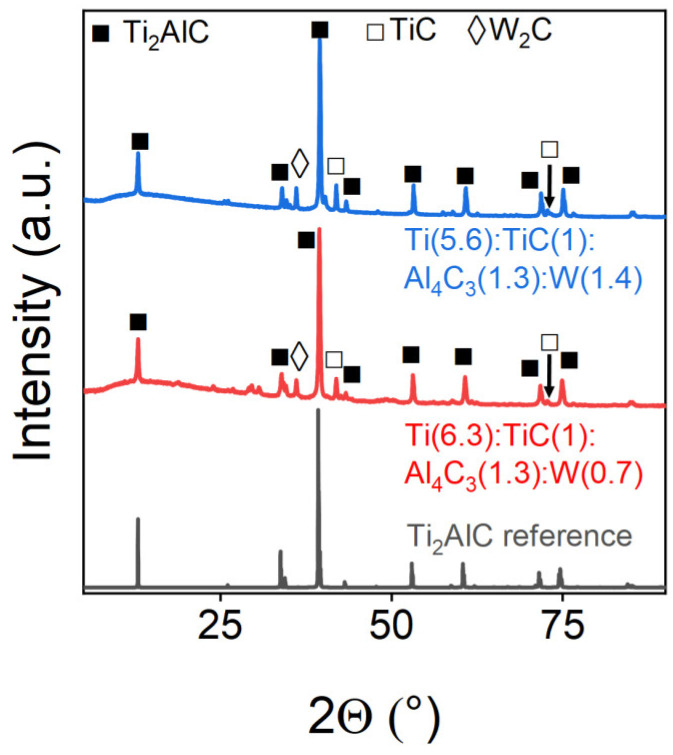
Powder X-ray diffraction patterns of W-doped samples. The marks above the reflexes indicate the corresponding target and impurity phases.

Tungsten demonstrated successful incorporation into the Ti_2_AlC MAX-phase lattice, as evidenced by the PXRD analysis (Figure 8) and Rietveld refinement (Table 6). Tungsten incorporation facilitated the formation of the titanium carbide secondary phase, which hinders the formation.

**Figure 9:**
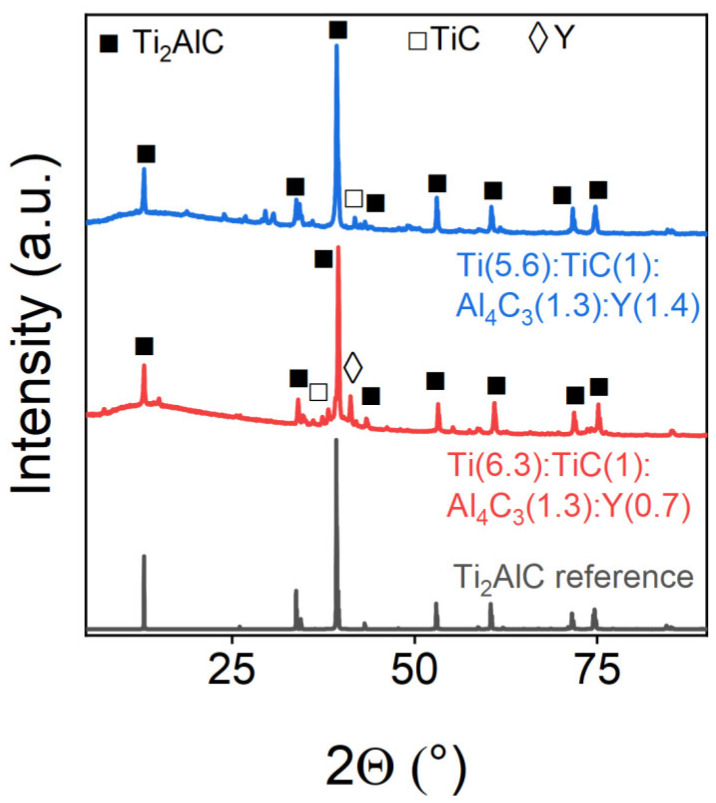
Powder X-ray diffraction patterns of Y-doped samples. The marks above the reflexes indicate the corresponding target and impurity phases.

Yttrium demonstrated successful incorporation into the MAX-phase structure, as evidenced by the PXRD patterns (Figure 9) and quantitative Rietveld refinement analysis (Table 7). The phase purity systematically improved with increasing the yttrium content, mirroring the beneficial doping effects previously observed for tantalum. This similarity suggests that both elements may enhance MAX-phase stability through comparable mechanisms during synthesis.

**Figure 10:**
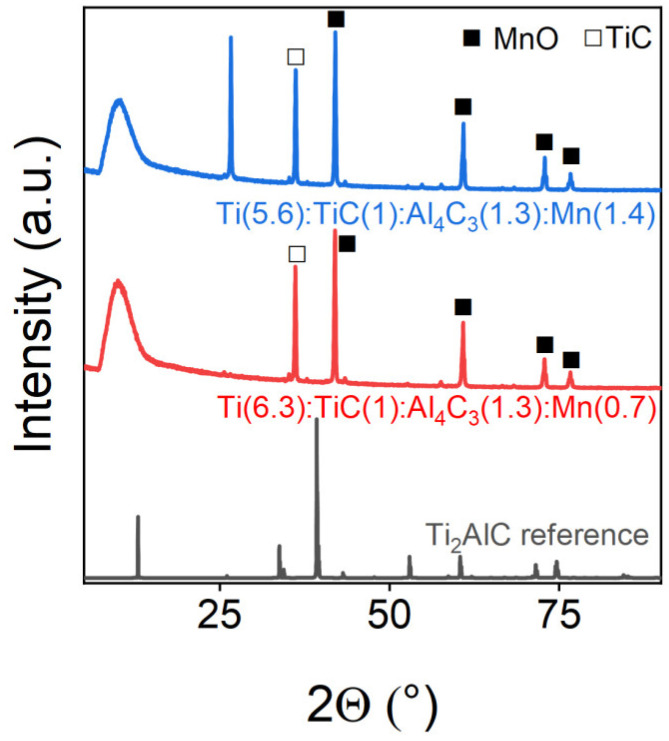
Powder X-ray diffraction patterns of Mn-doped samples. The marks above the reflexes indicate the corresponding target and impurity phases.

Manganese, when introduced into the MAX phase of Ti_2_AlC, did not allow the target phase to form at all, which is clearly visible in the PXRD, as shown in Figure 10. Thus, there was no main peak of Ti_2_AlC in the 40° region, while after sintering using the SPS method, titanium carbide with magnesium oxide was obtained, according to the results of XRD. The phase composition of the samples is shown in Table 8.

Table 9 summarizes the data on the optimal synthesis conditions for both pure 312 MAX phase by the SPS method and samples alloyed with various metals.

The obtained data indicate that Mo and Mn could not be incorporated into the Ti_2_AlC phase, while Ta, Hf, W, and Y efficiently substituted Ti atoms in the obtained (Ti_2−x_TM_X_)AlC (TM = Ta, Hf, W, Y). Substitution possibility depended on the mismatch between the radii of the host and substituent ions [21]. However, there were additional factors affecting the stability of the resulting compound, such as valence electron concentration [22] and thermodynamic stability [23]. An analysis of the activation and phase transformation processes is typically required for the assessment of the possibility of substituted phases [23]. We hope that the current work will pave the way for the computational analysis of TM-substituted MAX phases.

## 4. Conclusions

This study demonstrates that high-purity Ti_2_AlC MAX-phase ceramics can be synthesized via spark plasma sintering (SPS) by optimizing the precursor stoichiometry and thermal conditions. A 30 mol. % excess of Al_4_C_3_ beyond stoichiometric ratios required for Ti_3_AlC_2_ formation and a sintering temperature of 1300 °C were identified as optimal parameters, achieving over 90% Ti_2_AlC phase purity. Transition metal doping studies revealed distinct incorporation behaviors: Ta, Hf, W, and Y were successfully integrated into the Ti_2_AlC lattice, while Mo and Mn segregated as secondary phases. These findings advance the understanding of phase stability in doped MAX phases, providing critical guidelines for tailoring these materials toward structural and functional applications. The observed doping-dependent phase evolution underscores the importance of element-specific selection in MAX-phase design.

## Figures and Tables

**Table 1:** Elemental composition of the samples synthesized at various sintering temperatures.

Sample	Atomic Content, at. %			Ti:Al:C Ratio (Normalized to Aluminium)
	Ti	Al	C	
Ti(7):TiC(5):Al_4_C_3_(1)-1200	46.13 ± 0.09	11.71 ± 0.03	42.16 ± 0.10	3.94:1:3.6
Ti(7):TiC(5):Al_4_C_3_(1)-1300	48.09 ± 0.09	14.72 ± 0.03	37.20 ± 0.10	3.27:1:2.53
Ti(7):TiC(5):Al_4_C_3_(1)-1400	47.53 ± 0.09	12.70 ± 0.03	39.77 ± 0.10	3.74:1:3.13

**Table 2:** Phase composition of samples depending on the content of Al_4_C_3_.

Sample	Ti_2_AlC, wt.%	TiC, wt.%	AlTi_3_, wt.%	Ti_3_AlC_2_, wt.%
Ti(7):TiC(1):Al_4_C_3_(1.1)	86.18	2.48	11.34	-
Ti(7):TiC(1): Al_4_C_3_(1.3)	90.32	4.17	5.51	-
Ti(7):TiC(1):Al_4_C_3_ (1.5)	70.22	1.62	-	28.16

**Table 3:** Phase composition of Mo-doped samples.

Sample	Ti_2_AlC, wt.%	TiC, wt.%	AlTi_3_, wt.%	Mo, wt.%
Ti(6.3):TiC(1):Al_4_C_3_(1.3):Mo(0.7)	79.6	3.4	8.7	8.3
Ti(5.6):TiC(1):Al_4_C_3_(1.3):Mo(1.4)	71.11	14.43	-	14.46

**Table 4:** Phase composition of Ta-doped samples.

Sample	Ti_2_AlC, wt.%	TiC, wt.%	Ta, wt.%	Ti_3_AlC_2_, wt.%
Ti(6.3):TiC(1):Al_4_C_3_(1.3):Ta(0.7)	83.36	13.48	3.15	-
Ti(5.6):TiC(1):Al_4_C_3_(1.3):Ta(1.4)	89.06	8.56	2.39	-
Ti(4.9):TiC(1):Al_4_C_3_(1.3):Ta(2.1)	93.89	7.03	4.05	-
Ti(3.5):TiC(1):Al_4_C_3_(1.3):Ta(2.5)	45.62	2.77	0.61	51.00

**Table 5:** Phase composition of Hf-doped samples.

Sample	Ti_2_AlC, wt.%	TiC, wt.%	AlTi_3_, wt.%
Ti(6.3):TiC(1):Al_4_C_3_(1.3):HfC(0.7)	82.24	17.76	-
Ti(5.6):TiC(1):Al_4_C_3_(1.3):HfC(1.4)	90.51	-	9.49

**Table 6:** Phase composition of W-doped samples.

Sample	Weight Content of the Phase, %		
	Ti_2_AlC	TiC	W_2_C
Ti(6.3):TiC(1):Al_4_C_3_(1.3):W(0.7)	93.81	6.0	0.18
Ti(5.6):TiC(1):Al_4_C_3_(1.3):W(1.4)	91.61	7.9	0.49

**Table 7:** Phase composition of Y-doped samples.

Sample	Ti_2_AlC, wt.%	TiC, wt.%	Y, wt.%
Ti(6.3):TiC(1):Al_4_C_3_(1.3):Y(0.7)	88.91	4.36	6.74
Ti(5.6):TiC(1):Al_4_C_3_(1.3):Y(1.4)	97.79	2.21	-

**Table 8:** Phase composition of Mn-doped Ti_2_AlC samples.

Sample	MnO, wt.%	TiC, wt.%
Ti(6.3):TiC(1):Al_4_C_3_(1.3):Mn(0.7)	75.7	24.3
Ti(5.6):TiC(1):Al_4_C_3_(1.3):Mn(1.4)	61.5	38.5

**Table 9:** Optimal conditions for obtaining pure and alloyed Ti_2_AlC-based MAX phases.

Sample	Maximum Yeld of the Target Phase, %
Ti(7):TiC(1):Al_4_C_3_ (1.5)	70.22
Ti(7):TiC(1):Al_4_C_3_(1.1)	86.18
Ti(7):TiC(1):Al_4_C_3_(1.3)	90.32
Ti(7):TiC(1):Al_4_C_3_(1.3) doped by:	Maximum fraction of doping metal, mol.%
Mo	0
Ta	30
Hf	20
W	20
Y	20
Mn	0

## Data Availability

The original contributions presented in this study are included in the article/Supplementary Material. Further inquiries can be directed to the corresponding authors.

## Supplementary Materials

The following supporting information can be downloaded at: https://www.mdpi.com/article/10.3390/ma18091957/s1. Table S1: Sintering parameters of the samples obtained at various sintering temperatures. Table S2: Sintering parameters of the samples obtained at various aluminum excess. Table S3: Sintering parameters of Mo-doped samples. Table S4: Sintering parameters of Ta-doped samples. Table S5: Sintering parameters of Hf-doped samples. Table S6: Sintering parameters of W-doped samples. Table S7: Sintering parameters of Y-doped samples. Table S8: Sintering parameters of W-doped samples.
