# Supplementary material for: Spark Plasma Sintering of Pristine and Transition Metal-Doped Ti2AlC MAX Phases
Source: Materials (Basel). 2025 Apr 25;18(9):1957. doi: 10.3390/ma18091957 (PMC12072480; doi:10.3390/ma18091957)
Supplement: Supplementary file 1 [file materials-18-01957-s001.zip › materials-3557672-supplementary.pdf]

Spark plasma sintering of pristine and transition-metal-doped Ti_2AlC MAX-phase

Mikhail S. Gurin [1], Dmitry S. Shtarev [1,2,*], Ilya. A. Zavidovskiy [3], Erkhan S. Kolodeznikov [1], Andrey. A. Vyshnevyy [3,4], Aleksey V. Arsenin [3,4,5], Alexey D. Bolshakov [3,5,6,7] and Alexander V. Syuy [3,4,8,*]

[1] Department of Nuclear Technologies, Far Eastern Federal University, Vladivostok 690922, Russia; gurin.ms@dvfu.ru (M.S.G.); kolodeznikov.es@dvfu.ru (E.S.K.)
[2] Department of Materials Science, Shenzhen MSU-BIT University, Shenzhen 518115, China
[3] Moscow Center for Advanced Studies, Kulakova str. 20, Moscow 123592, Russia; ia.zavidovskii@physics.msu.ru (I.A.Z.), vyshnevyy@xpanceo.com (A.A.V.) arsenin@xpanceo.com (A.V.A.), bolshakov@live.com (A.D.B.)
[4] Emerging Technologies Research Center, XPANCEO, Dubai 00000, United Arab Emirates
[5] Laboratory of Advanced Functional Materials, Yerevan State University, Yerevan 0025, Armenia
[6] Center for Nanotechnologies, Alferov University, Saint Petersburg 194021, Russia
[7] Faculty of Physics, St. Petersburg State University, Saint Petersburg 199034, Russia
[8] Depatrment of Physics, Perm National Research Polytechnic University, Perm 614990, Russia
* Correspondence: shtarev@mail.ru (D.S.S.); alsyuy@xpanceo.com (A.V.S.)

Table S1. Sintering parameters of the samples obtained at various sintering temperatures.

Sample	Molar ratio			Sintering temperature, °C
	Ti	TiC	Al_4C_3	
Ti(7):TiC(5):Al_4C_3(1)-1200	7	5	1	1200
Ti(7):TiC(5):Al_4C_3(1)-1300	7	5	1	1300
Ti(7):TiC(5):Al_4C_3(1)-1400	7	5	1	1400

Table S2. Sintering parameters of the samples obtained at various aluminum excess.

Sample	Molar ratio			Sintering temperature, °C
	Ti	TiC	Al_4C_3	
Ti(7):TiC(1):Al_4C_3(1.1)	7	5	1.1	1300
Ti(7):TiC(1):Al_4C_3(1.3)	7	5	1.3	1300
Ti(7):TiC(1):Al_4C_3(1.5)	7	5	1.5	1300

Table S3. Sintering parameters of Mo-doped samples.

Sample	Molar ratio				Sintering temperature, °C
	Ti	TiC	Al_4C_3	Mo	
Ti(6.3):TiC(1):Al_4C_3(1.3):Mo(0.7)	6.3	5	1.3	0.7	1300
Ti(5.6):TiC(1):Al_4C_3(1.3):Mo(1.4)	5.6	5	1.3	1.4	1300

Table S4. Sintering parameters of Ta-doped samples.

Sample	Molar ratio				Sintering temperature, °C
	Ti	TiC	Al_4C_3	Ta	
Ti(6.3):TiC(1):Al_4C_3(1.3):Ta(0.7)	6.3	5	1.3	0.7	1300
Ti(5.6):TiC(1):Al_4C_3(1.3):Ta(1.4)	5.6	5	1.3	1.4	1300
Ti(4.9):TiC(1):Al_4C_3(1.3):Ta(2.1)	4.9	5	1.3	2.1	1300
Ti(3.5):TiC(1):Al_4C_3(1.3):Ta(2.5)	3.5	5	1.3	2.5	1300

Table S5. Sintering parameters of Hf-doped samples.

Sample	Molar ratio				Sintering temperature, °C
	Ti	TiC	Al_4C_3	HfC	
Ti(6.3):TiC(1):Al_4C_3(1.3):HfC(0.7)	6.3	5	1.3	0.7	1300
Ti(5.6):TiC(1):Al_4C_3(1.3):HfC(1.4)	5.6	5	1.3	1.4	1300

Table S6. Sintering parameters of W-doped samples.

Sample	Molar ratio				Sintering temperature, °C
	Ti	TiC	Al_4C_3	W	
Ti(6.3):TiC(1):Al_4C_3(1.3):W(0.7)	6.3	5	1.3	0.7	1300
Ti(5.6):TiC(1):Al_4C_3(1.3):W(1.4)	5.6	5	1.3	1.4	1300

Table S7. Sintering parameters of Y-doped samples.

Sample	Molar ratio				Sintering temperature, °C
	Ti	TiC	Al_4C_3	Y	
Ti(6.3):TiC(1):Al_4C_3(1.3):Y(0.7)	6.3	5	1.3	0.7	1300
Ti(5.6):TiC(1):Al_4C_3(1.3):Y(1.4)	5.6	5	1.3	1.4	1300

Table S8. Sintering parameters of W-doped samples.

Sample	Molar ratio				Sintering temperature, °C
	Ti	TiC	Al_4C_3	W	
Ti(6.3):TiC(1):Al_4C_3(1.3):Mn(0.7)	6.3	5	1.3	0.7	1300
Ti(5.6):TiC(1):Al_4C_3(1.3):Mn(1.4)	5.6	5	1.3	1.4	1300
